# Dietary Black Soldier Fly Larvae Meal Improves Growth Performance and Meat Quality Associated with Enhanced Antioxidant Capacity and Intestinal Health in Wenchang Chickens

**DOI:** 10.3390/ani16162608

**Published:** 2026-08-20

**Authors:** Xingke Wang, Qin Wang, Ruiying Bao, Qian Yang, Qingchao Yang, Zeru Peng, Yang Zhang, Haiwen Zhang, Huiyu Shi, Xuemei Wang

**Affiliations:** Animal Nutrition and Feed Laboratory, School of Tropical Agriculture and Forestry, Hainan University, Danzhou 571737, China; xkwang28@hainanu.edu.cn (X.W.); wangqin20210327@163.com (Q.W.); 23110710000036@hainanu.edu.cn (R.B.); 20233004953@hainanu.edu.cn (Q.Y.); 24220951330003@hainanu.edu.cn (Q.Y.); 22210905000001@hainanu.edu.cn (Z.P.); 25210905000015@hainanu.edu.cn (Y.Z.); hwzhang@hainanu.edu.cn (H.Z.); 993978@hainanu.edu.cn (H.S.)

**Keywords:** black soldier fly larvae meal, Wenchang chicken, meat quality, antioxidant capacity, intestinal health

## Abstract

Wenchang chicken, a premium indigenous yellow-feathered broiler in China, faces production constraints due to high feed costs and soybean meal dependence. Black soldier fly larvae meal (BSFLM) is a promising alternative protein source, but its effects on Wenchang chickens are largely unknown. This study evaluated the effects of dietary BSFLM on growth performance, meat quality, intestinal health, and cecal microbiota. A total of 192 fifty-day-old female Wenchang chickens were randomly assigned to diets with 0%, 5%, 10%, or 15% full-fat BSFLM for 42 days. The 15% BSFLM inclusion increased final body weight (*p* < 0.05) while reducing the feed conversion ratio (*p* < 0.01) and feed cost per kg gain (*p* < 0.001). Regarding meat quality, 15% BSFLM enhanced redness (a*) and reduced shear force, 48 h drip loss, and cooking loss (*p* < 0.05). It also elevated antioxidant enzyme activities, upregulated Nrf2–Keap1, improved intestinal villus architecture and tight junction proteins, and reduced serum diamine oxidase (*p* < 0.05). Cecal microbiota underwent distinct restructuring, with dose-dependent proliferation of beneficial taxa. Overall, BSFLM exerts beneficial effects on the feed efficiency, meat quality, and intestinal health of Wenchang chickens, and the 15% inclusion level achieved relatively better comprehensive outcomes. This study provides a scientific reference for insect protein utilization and contributes to sustainable high-quality Wenchang chicken production.

## Simple Summary

Wenchang chicken farmers face high feed costs and heavy dependence on soybean meal. Insect meal derived from black soldier fly larvae is a promising alternative protein, but its effects on this breed are unknown. We fed 192 female Wenchang chickens diets with 0%, 5%, 10%, or 15% insect meal for 42 days. The 15% insect meal diet increased final body weight, improved feed efficiency, and lowered the feed cost per kilogram of weight gain. Meat quality improved: it was redder, more tender, and retained more moisture. Gut health improved, with the results showing a better intestinal structure, healthier gut bacteria, a greater ability to fight cell damage, and reduced gut damage signs. Overall, replacing conventional feed with 15% insect meal has beneficial effects on the growth, meat quality, and gut health of Wenchang chickens. This study supports the use of insect protein to lower costs, reduce soybean reliance, and promote sustainable chicken production.

## 1. Introduction

As a globally consumed source of high-quality animal protein, chicken offers notable advantages, including a low carbon emission footprint and a well-balanced nutritional profile. The proteins, essential amino acids, and minerals that it provides effectively meet human physiological requirements, underscoring its significant role in the human diet [1,2]. In response to evolving consumer demand for “high-quality, safe, and distinctive” poultry products, yellow-feathered broilers are increasingly favored over white-feathered broilers in the global market due to their superior meat texture and unique flavor, positioning them as a regionally distinctive poultry breed with competitive advantages. Although the large-scale promotion of fast-growing poultry varieties—such as white-feathered Wenchang chickens and spent laying hens—has substantially pushed the physiological limits of growth rate and improved rearing efficiency, it has also led to notable declines in meat quality and flavor, including reduced tenderness and lower concentrations of flavor-related compounds, thereby failing to meet consumer expectations for high-quality poultry meat [3,4].

Wenchang chicken, a typical high-quality local yellow-feathered breed in southern China, is highly regarded by the market for its tender meat and rich flavor. However, its production is challenged by high rearing costs, with reported feed-to-gain ratios of 3.61:1 for males and 3.92:1 for females, and feed costs accounting for over 60% of total production expenses [5]. These high levels of feed consumption and associated costs have become critical bottlenecks limiting the large-scale and efficient production of Wenchang chicken. To address this challenge, the development of novel feed ingredients and the exploration of effective and efficient feed alternatives have emerged as key priorities for the sustainable development of the Wenchang chicken industry [6].

Protein ingredients derived from insects have garnered considerable interest as emerging alternatives for livestock and poultry feed formulations. The black soldier fly, in particular, represents a compliant and promising substitute that aligns effectively with the nutritional requirements of Wenchang chicken production. On a dry matter basis, this ingredient contains approximately 40–50% crude protein and exhibits a well-balanced amino acid composition, thereby constituting a viable replacement for conventional protein feedstuffs such as fishmeal and soybean meal and contributing to reduced reliance on traditional protein sources [7,8,9]. Furthermore, black soldier fly larvae are a natural reservoir of bioactive constituents—including antimicrobial peptides and lauric acid—which have been associated with improvements in host immune competence, a characteristic consistent with contemporary shifts toward antibiotic-free rearing practices. An additional advantage lies in the capacity to rear black soldier fly larvae on various organic waste streams, such as kitchen refuse and livestock excreta, thus facilitating waste bioconversion while simultaneously lowering feed input expenditures. This strategy yields concomitant economic and ecological benefits. The safety and practical feasibility of black soldier fly-derived feed materials have received preliminary corroboration through established industry benchmarks, thereby reinforcing their prospective role as an alternative dietary component for Wenchang chickens [10,11].

We hypothesized that dietary supplementation with graded levels of black soldier fly larvae meal (BSFLM) would exert dose-dependent beneficial effects on the growth performance, meat quality, and intestinal health of Wenchang chickens, and that these beneficial effects may be mediated by enhanced antioxidant capacity, improved intestinal barrier function, and modulated cecal microbiota composition. The present investigation examines the effects of graded dietary BSFLM concentrations on the growth performance, slaughter characteristics, meat quality attributes, and gastrointestinal health parameters of Wenchang chickens. Additionally, this study assesses the modulatory influence of BSFLM supplementation on muscular developmental traits, proximate nutrient composition, and the profile and abundance of intramuscular amino acids. Through 16S rRNA high-throughput sequencing, this work delineates the impact of BSFLM on the cecal microbial consortia of Wenchang chickens and further explores its repercussions on intestinal morphological architecture and barrier integrity.

Overall, this work endeavors to establish an empirical foundation for the exploitation of novel insect-derived protein feedstocks and the mitigation of reliance on soybean meal within poultry production systems. Moreover, the findings are anticipated to provide theoretical support for the advancement of ecologically sustainable and health-conscious husbandry methodologies.

## 2. Materials and Methods

### 2.1. Experimental Materials and Basal Diet Preparation

The premix and constituent raw materials employed in the present trial were sourced from Hainan Xinjiyuan Technology Co., Ltd. (Haikou, China). Whole-fat BSFLM was procured from Shenzhen Sijifeng Industrial Co., Ltd. (Shenzhen, China). The formulation of the basal diet adhered to the nutrient specifications outlined in the Chinese Feeding Standard for Chicken (NY/T 33-2004) [12]. The experimental diets were subsequently prepared by the research team through partial replacement of soybean meal with whole-fat BSFLM at predetermined inclusion ratios, with the core formulation principle of maintaining consistent crude protein levels and minimal variation in metabolizable energy across all treatments. Specifically, the crude protein content was kept constant to achieve an isonitrogenous design. The metabolizable energy values ranged from 12.24 to 12.28 MJ/kg, showing only negligible differences among treatments. The amino acid profiles presented minor fluctuations with increasing BSFLM inclusion, while all essential amino acid contents satisfied the nutritional requirements of Wenchang chickens. For lipid content and fatty acid composition, gradual elevations were observed with increasing BSFLM supplementation levels; these are inherent attributes of full-fat BSFLM and constitute some of the key variables investigated in this study. Details of the proximate nutrient profile and amino acid composition of the BSFLM are provided in Table S1 and Table S2, respectively. The ingredient composition and corresponding nutrient concentrations of the experimental diets are depicted in Table S3.

### 2.2. Animals and Experimental Design

A total of 192 50-day-old female Wenchang chickens were used in this experiment. Each bird was individually weighed and tagged for identification. The experiment was conducted at the animal feeding facility of Hainan University (Danzhou, China). The birds were reared in cages with ad libitum access to feed and water, and the experimental room was maintained with adequate ventilation. Feed was provided twice daily at 08:00 and 17:00. Environmental conditions, including temperature and humidity, were managed in accordance with the Poultry Management Manual. The birds were assigned to four dietary regimens using a randomized complete block design, with initial body weight serving as the blocking criterion. Each treatment comprised six replicate pens, with eight birds housed per pen. The replicate pen was defined as the experimental unit for growth performance analysis. The control group (designated as H0) received the basal diet exclusively, whereas the three experimental groups were provided with the basal diet supplemented with whole-fat black soldier fly larvae meal at inclusion rates of 5% (H5), 10% (H10), and 15% (H15). These substitutions were formulated to replace soybean meal on an isonitrogenous basis. These inclusion levels were selected based on previous studies demonstrating beneficial effects within this concentration range, as well as practical nutritional considerations, since excessively high inclusion rates may lead to reduced feed intake or impaired nutrient utilization.

### 2.3. Growth Performance and Slaughter Performance Measurements

To ascertain the influence of the dietary interventions on the growth performance of the Wenchang chickens, individual body weights were recorded at the initiation (day 1) and termination (day 42) of the feeding trial. Throughout the experimental period, daily feed provision and residual feed quantities were documented for each replicate. From these measurements, the average daily gain (ADG), average daily feed intake (ADFI), and feed conversion ratio (F/G) were computed. Furthermore, the feed expenditure per unit of body weight gain was derived by integrating the calculated F/G values with the feed costs associated with each dietary phase.

Upon termination of the feeding trial, all birds were subjected to 12 h of fasting prior to weighing, blood collection, and slaughter. The following parameters were recorded for each carcass: carcass weight, eviscerated weight (inclusive of edible giblets), breast muscle weight, leg muscle weight, and abdominal fat weight. In accordance with the guidelines outlined in the Terminology and Statistical Methods for Poultry Production Performance, dressing percentage, eviscerated yield, breast muscle yield, leg muscle yield, and abdominal fat percentage were derived using the measured values. The corresponding calculation formulae are defined as follows:

Feed cost per unit weight gain = average feed price × F/G ratio(1)

Dressing percentage (%) = (carcass weight/live weight before slaughter) × 100(2)

Eviscerated yield (%) = (eviscerated weight/live weight before slaughter) × 100(3)

Breast muscle yield (%) = (breast muscle weight/eviscerated weight) × 100(4)

Leg muscle yield (%) = (leg muscle weight/eviscerated weight) × 100(5)

Leg muscle yield (%) = (leg muscle weight/eviscerated weight) × 100(6)

Abdominal fat percentage (%) = (abdominal fat weight/eviscerated weight) × 100(7)

Wing yield (%) = 100 × wing weight/slaughter live weight(8)

### 2.4. Organ Index Measurement

To evaluate the influence of the dietary regimens on organ development in the Wenchang chickens, the kidneys, liver (following gallbladder excision), thymus, spleen, and bursa of Fabricius were harvested promptly after exsanguination, and their fresh weights were recorded. Organ indices were subsequently computed according to the following equation:

Organ index = fresh organ weight (g)/eviscerated weight (g)(9)

### 2.5. Measurement of Antioxidant Indicators in Serum, Muscle, and Intestine

Samples of muscle or intestinal tissue, weighing approximately 1 g, were homogenized with pre-cooled 0.9% physiological saline at a tissue-to-saline ratio of 1:9 (*w*/*v*) within an ice-water bath to yield tissue homogenates. Following centrifugation, the resultant supernatants were harvested for subsequent assays. Commercial detection kits (Nanjing Jiancheng Bioengineering Institute, Nanjing, China) were employed to quantify total antioxidant capacity (T-AOC), glutathione peroxidase (GSH-Px) activity, total superoxide dismutase (T-SOD) activity, and malondialdehyde (MDA) concentrations in both serum and tissue supernatant fractions. With respect to muscle specimens, the total protein content of the supernatant was determined using an Enhanced BCA Protein Assay Kit (Beyotime Biotechnology, Shanghai, China), thereby permitting the normalization of antioxidant enzyme activities to protein concentration on a per-unit-mass basis.

### 2.6. Meat Quality Measurement

Assessment of the meat quality attributes of the Wenchang chickens was conducted in compliance with the Agricultural Industry Standard of the People’s Republic of China—Objective Evaluation Method for Eating Quality of Meat (NY/T 2793-2015) [13]. One bird with body weight close to the replicate average was randomly selected from each replicate pen for slaughter sampling, resulting in six biological replicates per dietary treatment. All meat quality measurements were performed by the same well-trained operator to minimize systematic errors. Samples derived from the left breast muscle were utilized for the following determinations:

Drip loss: Breast muscle specimens were trimmed into uniformly dimensioned cubes, weighed to obtain initial mass, transferred into conical flasks, and stored upright under refrigeration at 4 °C. Specimens were reweighed following 24 h and 48 h intervals to compute the respective percentages of fluid exudation.

Cooking loss: Homogeneous breast muscle portions were weighed, appropriately labeled, placed within an aluminum vessel, and subjected to boiling in an induction cooker for a duration of 45 min. After being allowed to equilibrate to ambient temperature for 15 min, the samples were reweighed to determine the percentage of cooking-induced mass reduction.

Shear force: From each breast muscle sample, three strips measuring approximately 4.0 cm × 1.0 cm × 0.5 cm were excised in alignment with the orientation of the muscle fibers. Shear force was quantified using a muscle tenderness meter, with the blade applied perpendicular to the longitudinal axis of the fibers. Five replicate measurements were performed on each strip, and the mean value was documented.

Meat color: Chromatic parameters of both breast and leg musculature were recorded utilizing a 3NH colorimeter (Shenzhen, China). The instrument was calibrated with a standard white tile prior to each batch of measurements. The coordinates for lightness (L*), redness (a*), and yellowness (b*) were obtained.

pH value: Potentiometric measurements of pH were performed on breast and leg muscle samples at 45 min and 24 h postmortem using a calibrated pH meter. The pH meter was calibrated using standard buffer solutions (pH 4.0 and pH 7.0) prior to detection.

The corresponding computational formulae are defined as follows:

24 h drip loss (%) = 100 × (initial weight − weight after 24 h)/initial weight(10)

48 h drip loss (%) = 100 × (initial weight − weight after 48 h)/initial weight(11)

Cooking loss (%) = 100 × (pre-cooking weight − post-cooking weight)/pre-cooking weight(12)

### 2.7. Measurement of Conventional Nutrient Composition in Muscle

Quantification of proximate nutrient constituents within breast muscle specimens was performed in adherence to the prescribed protocols delineated in the National Food Safety Standards of the People’s Republic of China. Representative subsamples were excised from the left breast muscle of individual birds, subsequently pooled, and thoroughly homogenized to generate analytical samples. Moisture content was assessed in accordance with the National Food Safety Standard—Determination of Moisture in Foods (GB 5009.3-2016) [14]. Ash content was quantified following the specifications of the National Food Safety Standard—Determination of Ash in Foods (GB 5009.4-2016) [15]. Crude protein concentration was determined according to the guidelines established in the National Food Safety Standard—Determination of Protein in Foods (GB 5009.5-2016) [16]. Crude fat content was measured as stipulated by the National Food Safety Standard—Determination of Fat in Foods (GB 5009.6-2016) [17].

### 2.8. Determination of Fatty Acid and Free Amino Acid Composition in Muscle

Determination of free amino acid profiles and fatty acid constituents in the breast muscle tissues of the Wenchang chickens was carried out in accordance with previously established protocols [18]. Prior to compositional analysis, the collected muscle specimens were subjected to exhaustive desiccation using a freeze-drying apparatus.

The fatty acid composition was determined via gas–liquid chromatography employing an Agilent 7890A system (Santa Clara, CA, USA). Individual chromatographic peaks were assigned by matching retention times against authentic fatty acid standard references, and the proportional abundance of each fatty acid species was derived through peak area normalization. From these data, aggregate values for saturated fatty acids (SFAs), monounsaturated fatty acids (MUFAs), and polyunsaturated fatty acids (PUFAs) were subsequently computed.

Quantification of the free amino acid content in muscle tissue was accomplished utilizing an automated amino acid analyzer (Hitachi L-8900, Tokyo, Japan). The obtained measurements were normalized and reported as milligrams of amino acids per gram of fresh muscle mass (mg/g fresh weight).

### 2.9. Western Blot Analysis

Western blotting was employed to assess the abundance of target proteins in muscle and intestinal tissue specimens. In brief, aliquots of muscle or intestinal tissue were subjected to pulverization in liquid nitrogen, after which the total protein was isolated utilizing a tissue protein extraction kit (Servicebio, Wuhan, China). The resultant protein concentrations were measured with a BCA protein assay kit (Servicebio, Wuhan, China), and all lysates were equilibrated to uniform concentrations through the addition of extraction buffer. Equivalent quantities of protein were resolved electrophoretically on 10% SDS-PAGE gels and subsequently electro-transferred onto 0.45 μm polyvinylidene difluoride (PVDF) membranes. Upon completion of transfer, the membranes were blocked for 1 h at ambient temperature in a blocking solution composed of 5% non-fat milk. Thereafter, the membranes were probed overnight at 4 °C with the following panel of primary antibodies: rabbit anti-Keap1 polyclonal antibody (1:2000; Bioss, Beijing, China), rabbit anti-Nrf2 polyclonal antibody (1:2000; Bioss, Beijing, China), rabbit anti-Claudin-1 polyclonal antibody (1:2000; Bioss, Beijing, China), rabbit anti-Occludin polyclonal antibody (1:2000; Bioss, Beijing, China), rabbit anti-ZO-1 polyclonal antibody (1:2000; Bioss, Beijing, China), and mouse anti-β-actin monoclonal antibody (1:5000; Bioss, Beijing, China). All primary antibody solutions were prepared in antibody dilution buffer (Servicebio, Wuhan, China) consisting of 0.01 M Tris-HCl (pH 7.0–7.4) supplemented with 3% bovine serum albumin (BSA) and 0.1% Tween-20. The following day, the membranes were incubated for 1 h at room temperature with species-appropriate horseradish peroxidase-conjugated secondary antibodies (1:10,000; Bioss, Beijing, China). Immunoreactive bands were visualized using an automated chemiluminescence imaging system (Tanon 5200; Shanghai Tanon Science & Technology Co., Ltd., Shanghai, China), and densitometric analysis was performed with ImageJ software (version 1.54g). The relative expression levels of the target proteins were calculated as the ratio of the respective band intensity to that of the internal loading control (β-actin).

### 2.10. Histomorphology Analysis of Muscle and Intestinal Tissues

Histomorphometry evaluation of the breast muscle, leg muscle, and intestinal tissues of the Wenchang chickens was conducted in accordance with conventional histological processing techniques. Immediately following exsanguination, tissue specimens were harvested from the breast muscle; leg muscle; and medial portions of the duodenum, jejunum, and ileum. These specimens were rinsed gently with pre-cooled physiological saline to eliminate luminal contents and were promptly immersed in a 4% paraformaldehyde fixative solution.

Following the completion of fixation, the intestinal specimens underwent sequential dehydration through a series of ethanol solutions of progressively increasing concentration, were cleared with xylene, and were subsequently infiltrated with molten paraffin wax in accordance with standard histological processing procedures. Tissue sections were prepared at a designated thickness of 5 μm. These sections were then subjected to deparaffinization in xylene, rehydration via immersion in a descending graded ethanol sequence, and routine staining with hematoxylin and eosin (H&E). In brief, the tissue sections were immersed in hematoxylin solution, differentiated in acidified alcohol, rinsed under running tap water to achieve bluing, and subsequently counterstained with eosin solution. Following the staining procedure, the sections were dehydrated through a graded ethanol series, cleared in xylene, and cover-slipped using neutral mounting medium.

Stained histological sections were examined and digitally captured under bright-field microscopy. Morphometric parameters, specifically villus height and crypt depth, were quantified utilizing ImageJ software (version 1.54g). For each tissue section, a minimum of ten intact, longitudinally oriented intestinal villi were randomly selected for measurement, and the mean values derived from these determinations were employed for subsequent statistical analyses.

### 2.11. Determination of Intestinal Biochemical Parameters

Determination of digestive enzyme activities within the duodenal, jejunal, and ileal segments was performed according to the following protocol: Intestinal tissue specimens were retrieved from storage at −80 °C and gradually thawed on ice. Following precise gravimetric quantification, each sample was transferred into a pre-identified centrifuge tube and combined with ice-cold physiological saline at a tissue-to-saline ratio of 1:9 (*w*/*v*), after which sterile steel grinding beads were introduced. The sealed tubes were subsequently positioned within a refrigerated tissue homogenizer and subjected to thorough trituration at 4 °C to yield 10% (*w*/*v*) tissue homogenates. The resulting homogenates were centrifuged at 2500 rpm for 10 min under refrigerated conditions (4 °C). The clarified supernatants were then meticulously harvested, dispensed into aliquots, and stored at −80 °C pending subsequent analytical determinations.

The total protein concentration in the tissue supernatants was quantified using a BCA protein assay kit (Service bio, Wuhan, China). The activities of key digestive enzymes, including trypsin, chymotrypsin, amylase, and lipase, were determined using commercial assay kits (Nanjing Jiancheng Bioengineering Institute, Nanjing, China). Enzyme activities were expressed as units per milligram of protein (U/mg prot).

### 2.12. Cecal Microbiota Analysis

Compositional analysis of the cecal microbiota of the Wenchang chickens was conducted via 16S rRNA gene sequencing. Six individuals per experimental group were randomly selected, and genomic DNA was isolated from the cecal digesta specimens. The integrity of the extracted DNA was verified by electrophoresis on a 1% agarose gel. Subsequently, targeted amplification of the sequencing region was carried out using primers that were either barcoded with unique identifier sequences or designed as fusion primers incorporating deliberate base mismatches to enhance specificity. Representative samples were selected for preliminary experiments to ensure that the majority of samples could be amplified with appropriate product concentrations under minimal amplification cycles. PCR products were examined by 1% agarose gel electrophoresis and purified using an AxyPrep™ Mag PCR Clean-Up Kit. Libraries were then constructed and subjected to high-throughput sequencing on a next-generation sequencing platform.

To safeguard the fidelity and robustness of the sequencing output, raw sequence reads underwent a comprehensive quality control pipeline encompassing sequence assembly, stringent filtration, and the elimination of chimeric artifacts, thereby yielding a curated set of high-confidence sequences. Operational taxonomic units (OTUs) were subsequently delineated via clustering and assigned taxonomic identities through reference database alignment. Leveraging these clustering outcomes, assessments of both alpha diversity and beta diversity metrics were conducted. The resulting taxonomic assignments further permitted the retrieval of hierarchical classification information across multiple phylogenetic levels, thereby enabling comparative evaluations of community compositional profiles and the elucidation of inter-group variations in microbial community architecture.

### 2.13. Statistical Analysis

Statistical analyses were performed utilizing IBM SPSS Statistics (version 27), and graphical outputs were generated using GraphPad Prism (version 9). Prior to parametric analysis, the normality of the data distribution and homogeneity of variance were verified using the Shapiro–Wilk test and Levene’s test, respectively. All phenotypic data met the assumptions of one-way analysis of variance (ANOVA), and Tukey’s honestly significant difference (HSD) post hoc test was consistently applied for all pairwise multiple comparisons among dietary groups.

Alpha diversity indices were computed employing mothur software, and intergroup disparities in diversity were evaluated through the Wilcoxon rank-sum test, with the Benjamini–Hochberg procedure applied to control the false discovery rate (FDR) for multiple testing. Principal coordinate analysis (PCoA), derived from Bray–Curtis dissimilarity matrices and coupled with permutational multivariate analysis of variance (PERMANOVA), was conducted to identify significant structural divergences in microbial community composition among the experimental groups. Furthermore, linear discriminant analysis effect size (LEfSe) methodology (LDA score threshold > 2, *p* < 0.05) was applied to identify bacterial taxa exhibiting statistically significant differential relative abundances across taxonomic hierarchies ranging from the phylum to the genus level.

## 3. Results

### 3.1. Effects of Dietary BSFLM on Growth Performance and Slaughter Performance of Wenchang Chickens

As shown in Table 1, incorporation of BSFLM into the diet was found to enhance the growth performance of Wenchang chickens. Notably, birds in the H15 group attained a significantly greater final body weight than those in the H0 control group (*p* < 0.05). Across the three BSFLM-supplemented groups, the average daily gain (ADG) exhibited an increase and the average daily feed intake (ADFI) showed a slight decline relative to the H0 group; however, these alterations did not reach statistical significance (*p* > 0.05). Additionally, the feed conversion ratio (F/G) was reduced in all treatment groups, with the high-dose H15 group showing a statistically significant improvement (*p* < 0.05). Furthermore, dietary supplementation with BSFLM resulted in a marked decrease in feed expenditure per unit of body weight gain compared with the H0 regimen (*p* < 0.05), and this reduction was particularly pronounced in the H15 group, reaching a high level of statistical significance (*p* < 0.01).

As shown in Table 2, the inclusion of graded concentrations of BSFLM in the diet did not cause any statistically significant alterations in pre-slaughter live weight; dressing percentage; breast muscle yield; leg muscle yield; eviscerated yield; wing yield; abdominal fat percentage; or the relative organ weights of the liver, thymus, spleen, bursa of Fabricius, and kidneys (*p* > 0.05).

**Figure 1:**
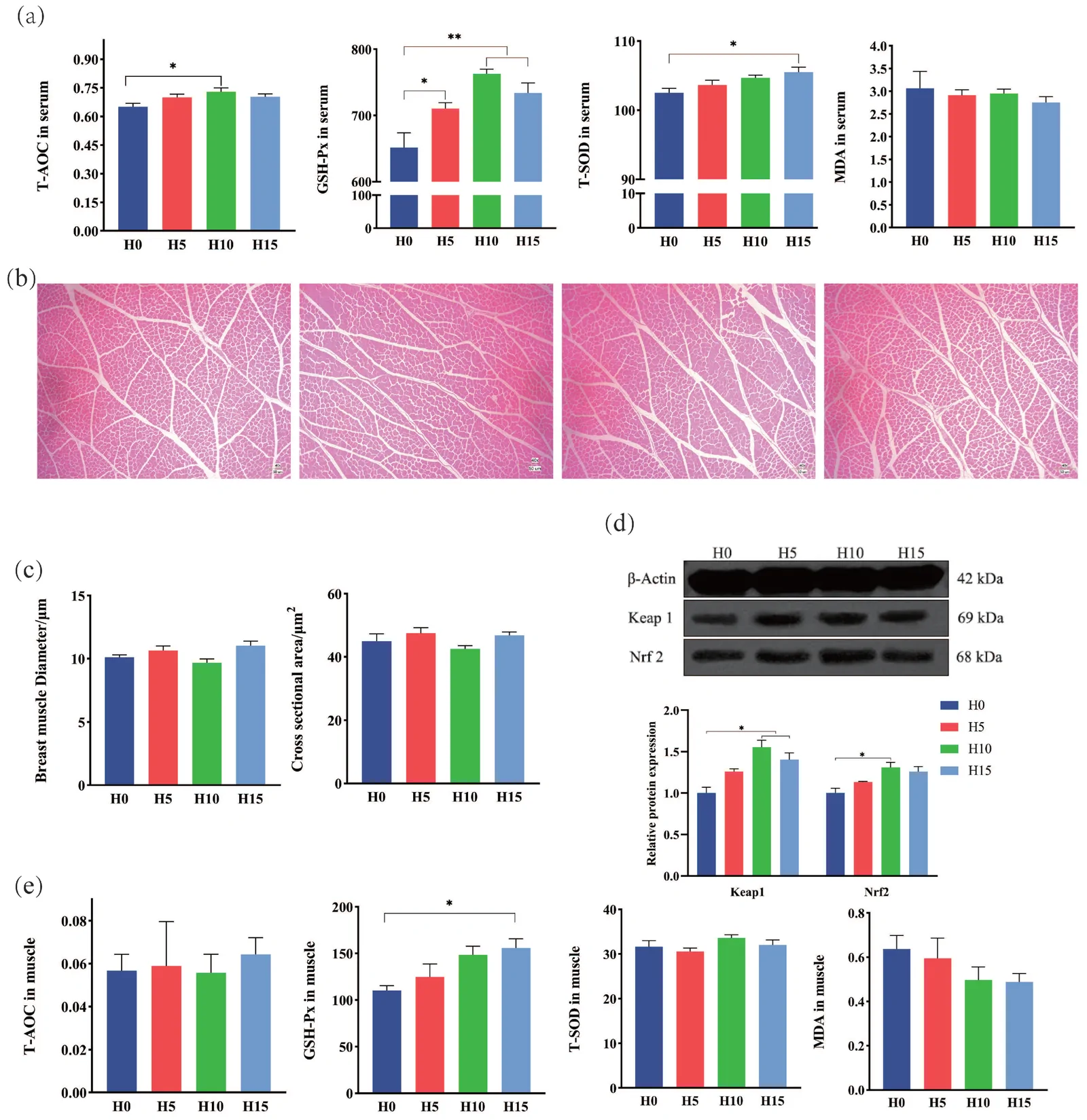
Effects of dietary BSFLM on muscle traits of Wenchang chickens. (**a**) Serum antioxidant enzyme-related indicators; *n* = 6. (**b**) Representative images of breast H&E staining. (**c**) Muscle fiber diameter and cross-sectional area; *n* = 3. (**d**) Expression levels of Keap1 and Nrf2 proteins in the pectoral muscles; *n* = 3. (**e**) Breast antioxidant enzyme-related indicators; *n* = 6. Values and vertical lines are means ± SEM. * *p* < 0.05, ** *p* < 0.01.

Meanwhile, the results of the serum antioxidant parameters of the Wenchang chickens are presented in Figure 1a. Compared with the H0 group, the H5 group exhibited increased serum levels of T-SOD and T-AOC, although the differences were not statistically significant (*p* > 0.05); however, the H5 group showed significantly increased serum GSH-Px activity (*p* < 0.05). Both the H10 and H15 groups showed highly significant increases in serum GSH-Px levels (*p* < 0.01). Additionally, the H10 group showed significantly elevated serum T-AOC (*p* < 0.05), and the H15 group showed significantly increased serum T-SOD activity (*p* < 0.05). No significant differences were observed in serum MDA levels among the treatment groups (*p* > 0.05).

### 3.2. Meat Quality of Wenchang Chickens

The effects of dietary supplementation with different levels of BSFLM on the meat quality traits of Wenchang chickens are presented in Table 3. Compared with the H0 group, the H15 group exhibited a significant increase in meat redness (a* value), which may indicate a higher myoglobin content in the muscle and is generally considered indicative of fresh, high-quality meat. Furthermore, the shear force values showed a dose-dependent decreasing trend with increasing BSFLM inclusion, and a significant reduction was observed only in the 15% BSFLM group compared with the control. Both 48 h drip loss and cooking loss exhibited statistically significant reductions (*p* < 0.05), findings that collectively point toward enhanced muscle tenderness and improved water-holding capacity. In contrast, no appreciable intergroup variations were detected for lightness (L* value), yellowness (b* value), pH measurements, or drip loss assessed at the 24 h interval (*p* > 0.05). The influence of the graded dietary inclusion of BSFLM on the histomorphological characteristics of the breast muscle of Wenchang chickens is depicted in Figure 1b,c. Relative to the H0 control group, dietary supplementation with BSFLM did not exert any significant impact on either muscle fiber diameter or mean cross-sectional area (*p* > 0.05).

### 3.3. Effects of Dietary BSFLM on Muscle Antioxidant Capacity of Wenchang Chickens

The effects of dietary supplementation with different levels of BSFLM on the oxidative status-related parameters of the muscle of Wenchang chickens are shown in Figure 1e. Relative to the H0 control group, both the H10 and H15 treatment groups showed lower muscle malondialdehyde (MDA) concentrations; nevertheless, these reductions failed to reach statistical significance (*p* > 0.05). Concurrently, glutathione peroxidase (GSH-Px) activity in muscle tissue was elevated across the supplemented groups, with the H15 high-dose group exhibiting a statistically significant difference (*p* < 0.05). In contrast, no significant intergroup differences were evident in either total antioxidant capacity (T-AOC) or total superoxide dismutase (T-SOD) activity (*p* > 0.05).

The influence of the graded dietary inclusion of BSFLM on the abundance of proteins associated with the oxidative stress signaling pathway in the muscle tissue of Wenchang chickens is depicted in Figure 1d. Relative to the H0 control group, a marked upregulation in the protein expression levels of both Keap1 and Nrf2 was observed in the H10 treatment group (*p* < 0.05).

### 3.4. Effects of Dietary BSFLM on Amino Acid and Fatty Acid Profiles of Wenchang Chickens

The effects of dietary supplementation with different levels of BSFLM on the amino acid content of the breast muscle of Wenchang chickens are presented in Table 4. Relative to the H0 control group, birds assigned to the H10 treatment showed a statistically significant elevation in aspartic acid (Asp) concentration, whereas the H15 group exhibited pronounced increases in both methionine (Met) and leucine (Leu) contents (*p* < 0.05).

As shown in Table 5, the effects of the different treatment groups on the fatty acid composition of the breast muscle were primarily reflected in the contents of monounsaturated fatty acids (especially C18:1n9c) and C20:2 (n-6 PUFA). Specifically, relative to the H0 control group, birds in the H10 and H15 treatment groups showed significantly elevated concentrations of C18:1n9c (oleic acid) in breast muscle tissue (*p* < 0.05). The highest value was recorded in the H10 group (43.75), followed closely by the H15 group (43.37). In contrast, no statistically significant difference was detected between the H5 group and the control group (*p* > 0.05). With respect to the total monounsaturated fatty acids (MUFAs), both the H10 and H15 groups exhibited significantly greater levels than the H0 control (*p* < 0.05), whereas the H5 group did not differ appreciably from the control (*p* > 0.05). Furthermore, the concentration of C20:2 (an n-6 PUFA) in the H10 group was found to be significantly lower than that observed in the remaining treatment groups (*p* < 0.05), with no significant intergroup differences evident among the H0, H5, and H15 groups (*p* > 0.05). No significant differences were detected across the treatment groups for any of the other fatty acid constituents assayed or for the total saturated fatty acids (SFAs), total polyunsaturated fatty acids (PUFAs), and unsaturated-to-saturated fatty acid (USFA/SFA) ratio (*p* > 0.05).

### 3.5. Effects of Dietary BSFLM on Intestinal Antioxidant Capacity and Digestive Function of Wenchang Chickens

**Figure 2:**
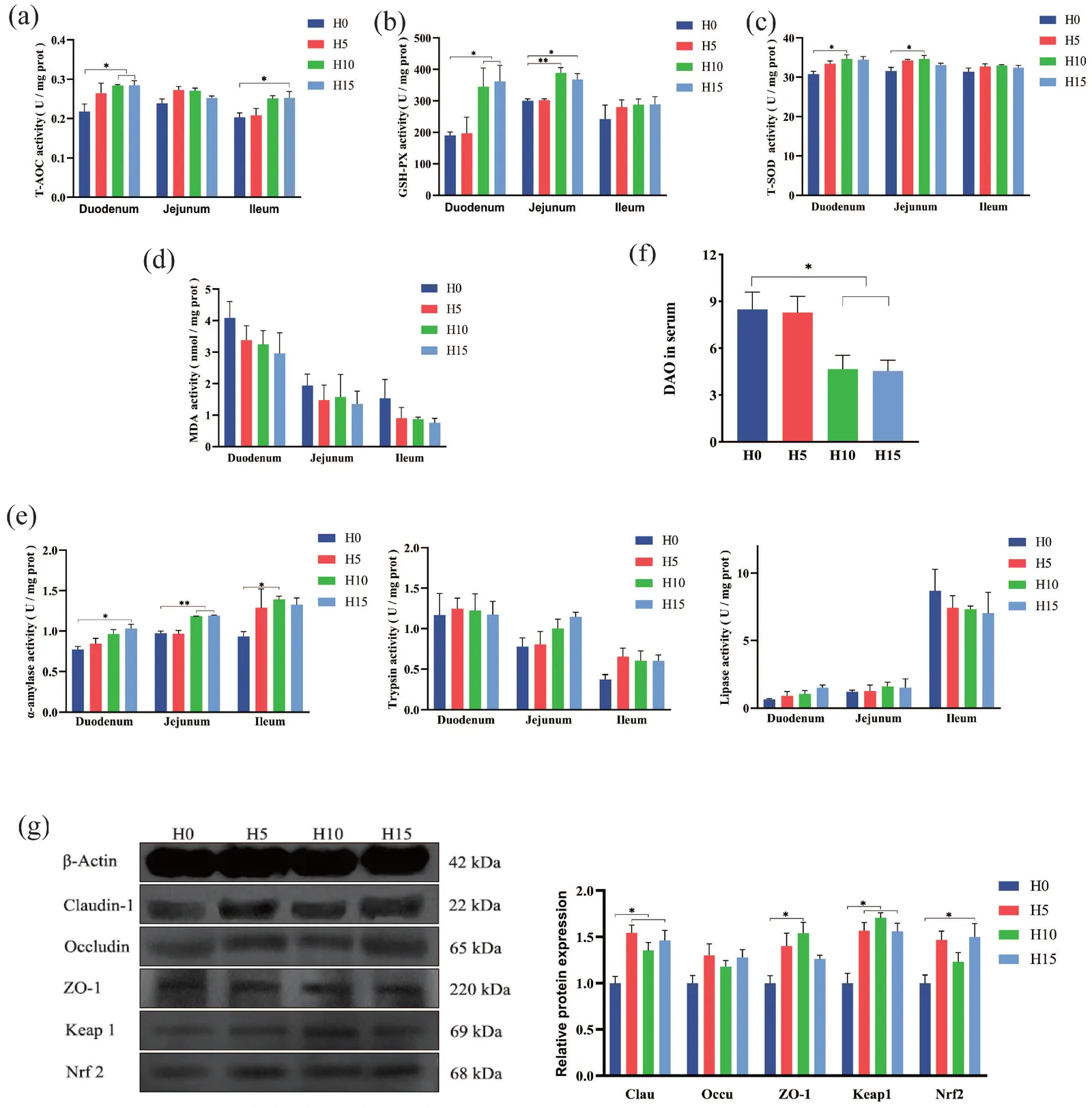
Effects of dietary BSFLM on intestinal indicators of Wenchang chickens. (**a**–**d**) Mucous membrane of small intestine antioxidant enzyme-related indicators; *n* = 6. (**e**) The levels of α-amylase, trypsin, and lipase in small intestine segment; *n* = 6. (**f**) The level of DAO; *n* = 6. (**g**) Expression levels of Claudin-1, Occludin, ZO-1, Keap1, and Nrf2 proteins in the mucous membrane of small intestine; *n* = 3. Values and vertical lines are means ± SEM. * *p* < 0.05, ** *p* < 0.01.

The effects of dietary supplementation with different levels of BSFLM on the oxidative status-related parameters of the small intestinal segment of Wenchang chickens are shown in Figure 2a–d. Compared with the H0 group, the H5 group showed increased activities of GSH-Px and T-SOD, elevated T-AOC, and decreased MDA levels in the duodenum, although none of these differences reached statistical significance. The H10 and H15 groups exhibited significantly increased GSH-Px activity (*p* < 0.05) and significantly elevated T-AOC (*p* < 0.05) in the duodenum, with no significant effects on MDA levels. Both groups also showed increased T-SOD activity, with a significant increase observed in the H10 group (*p* < 0.05). Compared with the H0 group, the H5 group showed increased GSH-Px and T-SOD activities in the jejunum, although these differences were not statistically significant (*p* > 0.05). In the jejunal tissue, the H10 treatment group showed a highly significant elevation in glutathione peroxidase (GSH-Px) activity (*p* < 0.01), accompanied by a statistically significant difference in total superoxide dismutase (T-SOD) activity (*p* < 0.05). In the H15 group, GSH-Px activity was also significantly increased (*p* < 0.05), whereas the rise in T-SOD activity failed to reach statistical significance (*p* > 0.05). No appreciable intergroup differences were detected in either the total antioxidant capacity (T-AOC) or malondialdehyde (MDA) concentrations in the jejunum across the experimental groups (*p* > 0.05). Turning to the ileal segment, birds receiving the H15 dietary regimen exhibited a significant enhancement in T-AOC relative to the H0 control group (*p* < 0.05). Across all groups supplemented with BSFLM, increases in both GSH-Px activity and T-AOC were noted, alongside a modest reduction in MDA content; nonetheless, these alterations did not reach the threshold of statistical significance (*p* > 0.05).

The effects of dietary supplementation with different levels of BSFLM on different digestive enzyme activities in various intestinal segments of Wenchang chickens are shown in Figure 2e. Compared with the H0 group, the H5 group showed no significant changes in α-amylase activity in the duodenum, jejunum, or ileum. In contrast, the H10 and H15 groups exhibited highly significant increases in α-amylase activity in the jejunum (*p* < 0.01). Additionally, the H10 group showed significantly increased α-amylase activity in the ileum (*p* < 0.05), while the H15 group showed significantly increased α-amylase activity in the duodenum (*p* < 0.05). The positive effects of BSFLM on α-amylase activity in different intestinal segments were positively correlated with the dietary inclusion level to some extent. Compared with the H0 group, the H5, H10, and H15 groups showed increased lipase activity in the duodenum, while the H10 and H15 groups showed increased lipase activity in the jejunum; however, none of these differences reached statistical significance (*p* > 0.05). Moreover, no significant differences in lipase activity were observed in the ileum among the treatment groups (*p* > 0.05). Although the inclusion of BSFLM exerted a certain enhancing effect on lipase activity across different intestinal segments, the effects were not statistically significant. Compared with the H0 group, the H5, H10, and H15 groups all showed increased trypsin activity in the duodenum, jejunum, and ileum, but these differences were not statistically significant (*p* > 0.05). The inclusion of BSFLM exhibited a certain enhancing effect on trypsin activity across different intestinal segments, although the effects were not significant.

### 3.6. Effects of Dietary BSFLM on Intestinal Barrier Function of Wenchang Chickens

The effects of dietary supplementation with different levels of BSFLM on serum diamine oxidase (DAO) activity, an indicator of intestinal permeability, are shown in Figure 2f. Compared with the H0 group, the H5 group showed no significant difference (*p* > 0.05), whereas the H10 and H15 groups exhibited significantly reduced serum DAO levels (*p* < 0.05). These results indicate that BSFLM supplementation decreases serum levels of the intestinal barrier marker DAO in Wenchang chickens.

The effects of dietary supplementation with different levels of BSFLM on the expression of intestinal barrier and antioxidant-related proteins are summarized in Figure 2g. Compared with the H0 group, the H5 and H15 groups exhibited significantly increased protein expression levels of ZO-1, Claudin-1, and Keap 1 (*p* < 0.05).

**Figure 3:**
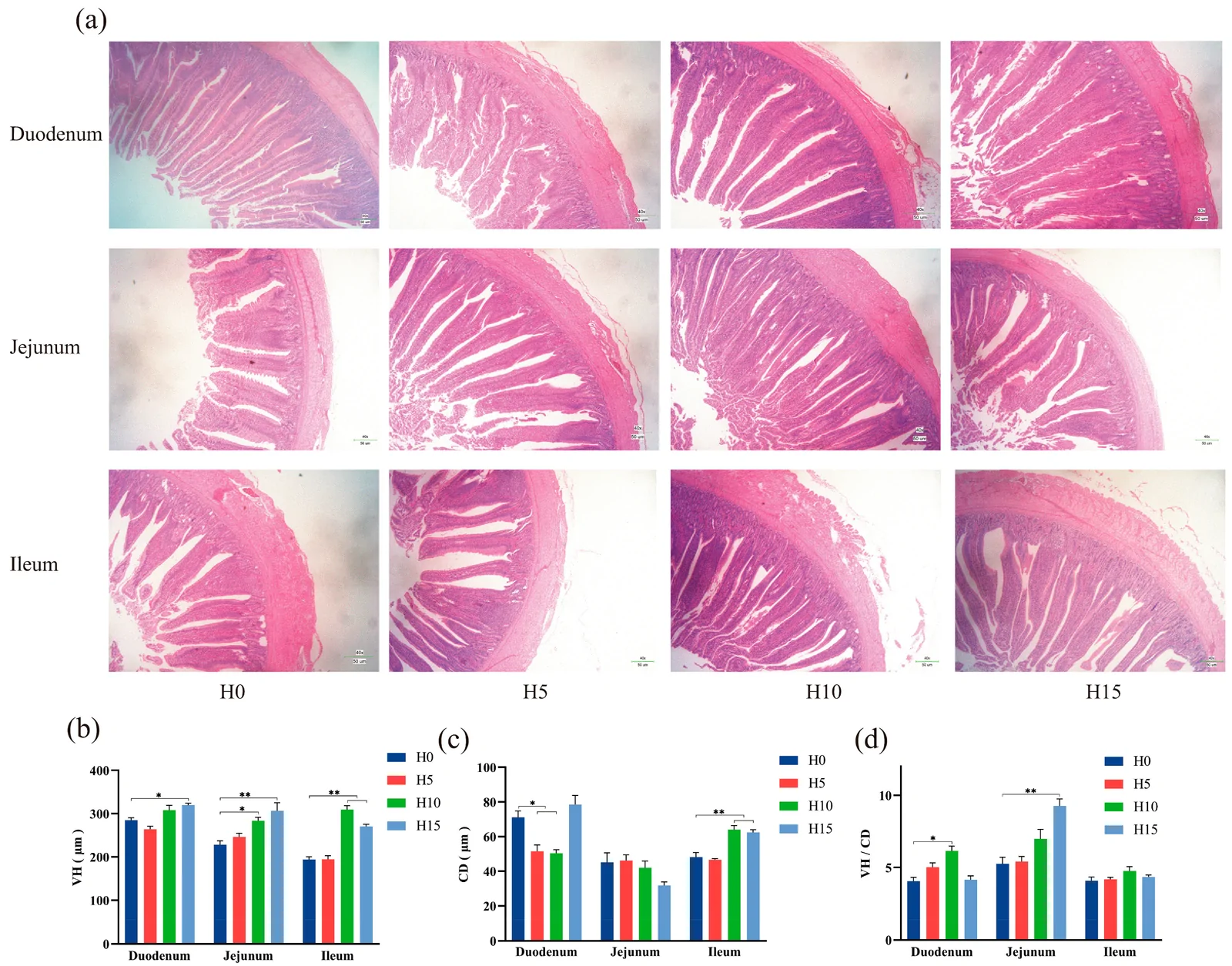
Effects of dietary BSFLM on intestinal morphology of Wenchang chickens. (**a**) Representative images of small intestine segment H&E staining. (**b**) Villus height (VH); *n* = 3. (**c**) Crypt depth (CD); *n* = 3. (**d**) Villus height-to-crypt depth ratio (VH/CD); *n* = 3. Values and vertical lines are means ± SEM. * *p* < 0.05, ** *p* < 0.01.

The effects of dietary supplementation with different levels of BSFLM on the small intestinal microstructure of Wenchang chickens are presented in Figure 3. In the duodenum, compared with the H0 group, the H15 group showed significantly increased villus height, and the H10 and H15 groups exhibited significantly increased villus height-to-crypt depth ratios, while the H5 group showed a significant decrease in crypt depth (*p* < 0.05). In the jejunum, the H15 group demonstrated a significantly increased villus height-to-crypt depth ratio compared with the H0 group (*p* < 0.05). In the ileum, compared with the H0 group, the H10 and H15 groups showed highly significant increases in villus height (*p* < 0.05), whereas no significant changes were observed in the villus height-to-crypt depth ratio (*p* > 0.05). Collectively, these findings suggest that BSFLM supplementation improves the intestinal morphological structure of Wenchang chickens.

### 3.7. Effects of Dietary BSFLM on Cecal Microbiota of Wenchang Chickens

**Figure 4:**
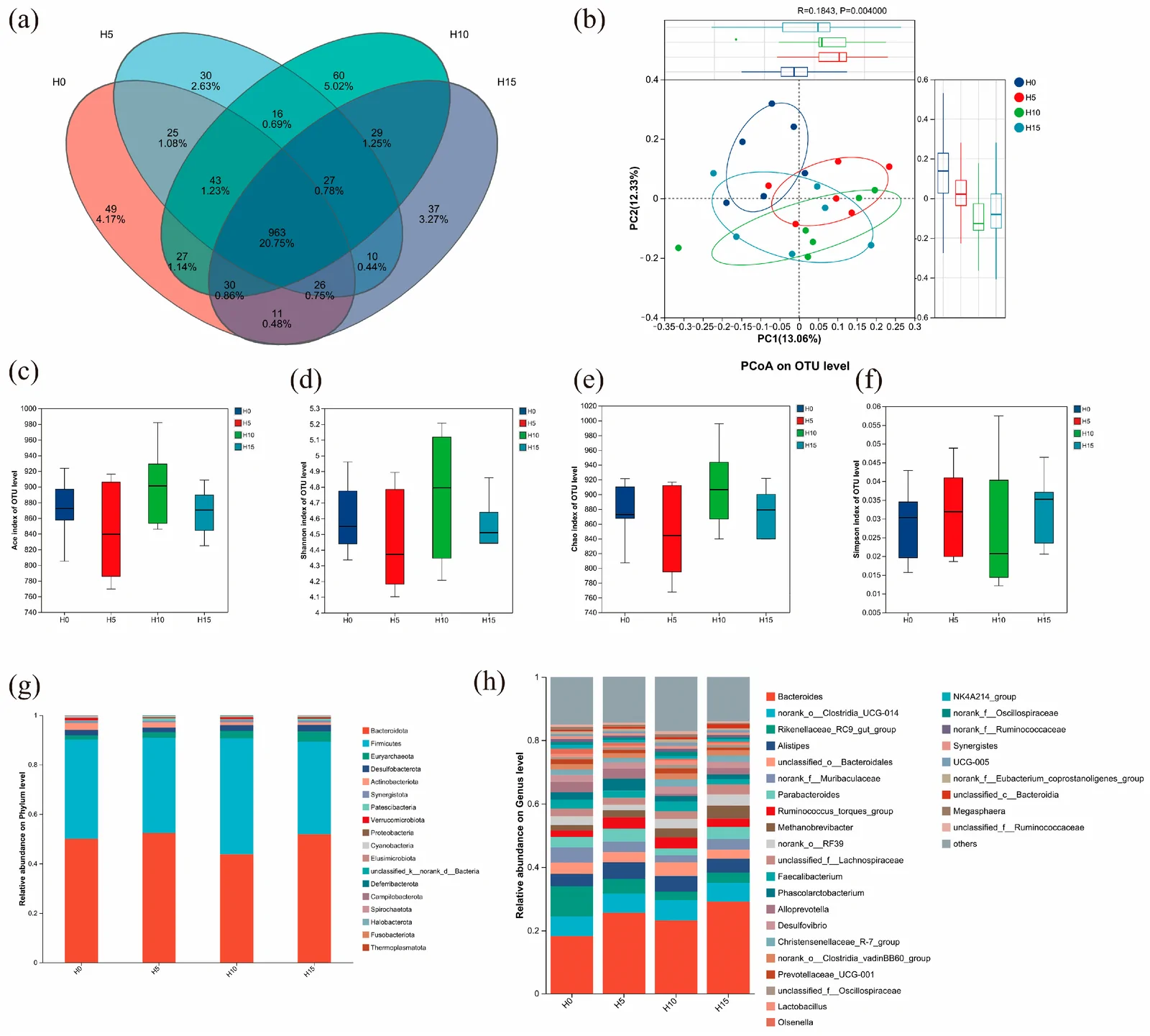

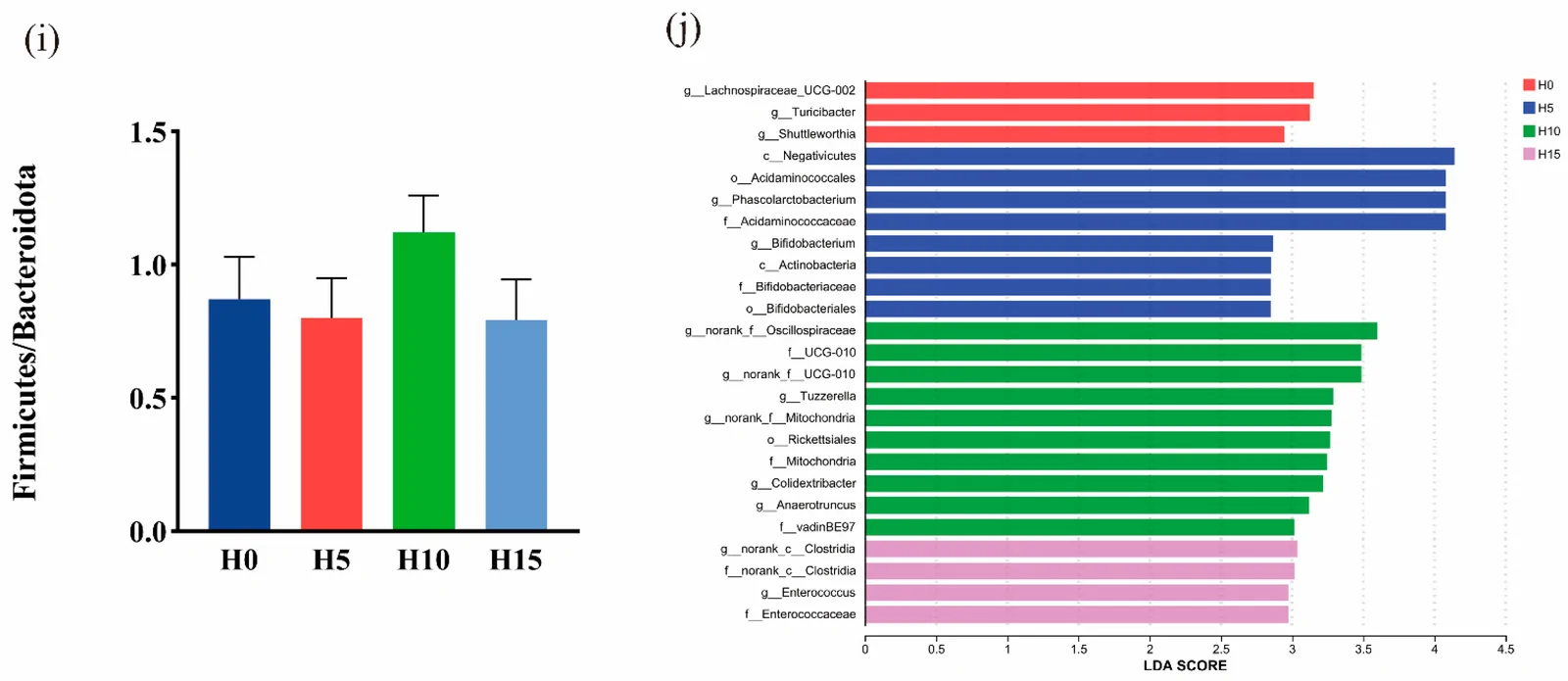
Effects of dietary BSFLM on cecal microbiota of Wenchang chickens. (**a**) Venn diagram of OUT distribution. (**b**) Beta diversity analysis of microbial communities; panels (**c**), (**d**), (**e**), and (**f**) show the alpha diversity indices (Ace, Shannon, Chao, and Simpson) of intestinal microbiota. (**g**) Microbiota composition at the phylum level. (**h**) Microbiota composition at the genus level. (**i**) Ratio of Firmicutes to Bacteroidetes. (**j**) LDA distribution. Values are means ± SEM. The bold horizontal lines in panels (**c**–**f**) indicate the median values.

16S rRNA amplicon sequencing of cecal contents revealed a total of 963 shared operational taxonomic units (OTUs) among the four groups, with 1174, 1140, 1195, and 1133 OTUs detected in the H0 (control), H5, H10, and H15 groups, respectively (Figure 4a).

Principal coordinate analysis (PCoA) based on Bray–Curtis distances was performed to assess the overall differences in microbial community structure (Figure 4b). A clear separation was observed between the H0 control group and all BSFLM-supplemented groups (PERMANOVA, *R* = 0.1843, *p* = 0.004), indicating that dietary BSFLM significantly altered cecal microbial composition. The H5, H10, and H15 groups showed overlapping distribution patterns, suggesting similar microbial community structures among the BSFLM-fed chickens.

Assessment of alpha diversity metrics, encompassing the Shannon, Simpson, ACE, and Chao indices, revealed no statistically significant differences in either microbial richness or community evenness when comparing any of the BSFLM-supplemented treatment groups with the H0 control (*p* > 0.05) (Figure 4c–f). These findings collectively demonstrate that the dietary incorporation of black soldier fly larvae meal exerted no discernible influence on the overall alpha diversity of the cecal microbiota of the Wenchang chickens.

At the phylum taxonomic level, Bacteroidota and Firmicutes were the predominant constituents across all experimental groups, jointly representing in excess of 88% of the total cecal microbial community. These were followed in relative abundance by Euryarchaeota, Desulfobacterota, and Actinobacteriota (Figure 4g). No statistically significant intergroup differences were observed in the proportional representation of these dominant phyla (*p* > 0.05). In accordance with this observation, the Firmicutes-to-Bacteroidota ratio—a widely recognized indicator of intestinal microbial equilibrium—exhibited no significant difference among the four dietary treatment groups (*p* > 0.05) (Figure 4i).

At the genus level, the core microbiota was dominated by *Bacteroides*, norank_o_Clostridia_UCG-014, Rikenellaceae_RC9_gut_group, *Alistipes*, unclassified_o_Bacteroidales, norank_f_Muribaculaceae, and *Parabacteroides* (Figure 4h). Linear discriminant analysis effect size (LEfSe) analysis identified 25 differentially abundant taxa (LDA score > 2) that drove the separation between groups (Figure 4j). Specifically, *Lachnospiraceae_UCG-002*, *Turicibacter*, and *Shuttleworthia* were enriched in the H0 control. The H5 group was characterized by significant enrichment of the beneficial genus *Bifidobacterium* (*p* < 0.05), along with Negativicutes, Acidaminococcales, and *Phascolarctobacterium*. The H10 group showed enrichment of Oscillospiraceae-related taxa, *Tuzzerella*, and *Colidextribacter*, while the H15 group was distinguished by increased abundances of Clostridia and *Enterococcus*.

## 4. Discussion

Wenchang chicken, a premium indigenous yellow-feathered broiler in China, faces production constraints due to high feed costs and soybean meal dependence. The demand for sustainable protein sources in poultry production has driven research into alternative feedstuffs that can replace soybean meal and fishmeal [19]. Black soldier fly larvae meal (BSFLM) has attracted considerable interest as a novel feed resource because of its favorable amino acid profile, high nutrient bioavailability, and relatively low ecological footprint. Previous studies have shown that poultry responses to BSFL products vary substantially with dietary inclusion level, processing method, and host genotype [8,20,21]. In the present study, we systematically evaluated the effects of graded levels of full-fat BSFLM (5%, 10%, and 15%) on the growth performance, antioxidant status, meat quality, intestinal health, and cecal microbiota of Wenchang chickens. Our findings offer both mechanistic insights and practical support for adopting BSFLM as a sustainable feed ingredient in Wenchang chicken production.

Dietary BSFLM exerted dose-dependent effects on growth performance. The 15% BSFLM group showed a significantly increased final body weight and a markedly reduced feed conversion ratio (F/G), while the other growth parameters did not differ significantly among groups. These results are partly consistent with those of previous reports showing that BSFL supplementation improved growth and lowered F/G in chicks [22], which can be ascribed to the high nutritional value of BSFLM. Full-fat BSFLM is abundant in crude fat, crude protein, and essential amino acids that promote muscle development, and it is highly digestible in broilers [23]. Importantly, the apparent absorption of crude fat from BSFL surpasses that of soybean meal [24], providing a plausible explanation for the improved feed efficiency observed. With respect to slaughter performance, BSFLM inclusion up to 15% did not significantly affect slaughter traits, in agreement with findings in broilers [25,26]. The 15% BSFLM group showed an increase in abdominal fat percentage, which may be related to the high fat content of full-fat BSFLM [27].

Oxidative stress constitutes a major constraint in poultry production because excessive reactive oxygen species (ROS) damage cellular DNA, proteins, and lipids, triggering apoptosis, immunosuppression, and intestinal inflammation, thereby compromising performance and product quality [28]. Our data demonstrated that BSFLM enhanced both systemic and intestinal antioxidant capacity, as evidenced by elevated serum total antioxidant capacity (T-AOC), total superoxide dismutase (T-SOD) activity, and glutathione peroxidase (GSH-Px) activity, along with upregulated antioxidant enzyme activities in the small intestinal mucosa. These observations align with those of previous reports showing that BSFLM increased plasma GSH-Px and T-SOD while decreasing malondialdehyde (MDA) in laying hens [29,30]. The antioxidant activity of BSFLM may be attributed to its bioactive constituents, including protein fractions with radical-scavenging ability and chitin [31]. To explore the underlying molecular mechanism, we investigated the Nrf2–Keap1 pathway, the master regulator of cellular antioxidant defense. BSFLM supplementation upregulated Nrf2 and Keap1 protein expression in breast muscle and the jejunum, concomitant with increased GSH-Px activity and reduced MDA levels. Under normal conditions, Nrf2 is retained in the cytoplasm by Keap1; upon oxidative insult, Nrf2 dissociates, translocates to the nucleus, and binds to antioxidant response elements, thereby driving the transcription of cytoprotective enzymes such as SOD and GSH-Px [32]. Thus, the improvements in growth performance and meat quality induced by BSFLM may be driven by multiple underlying mechanisms. In addition to activating the Nrf2-Keap1 signaling pathway and thereby strengthening redox homeostasis and inhibiting lipid and protein oxidation, the enhanced amino acid availability, naturally occurring antimicrobial peptides, and optimized fatty acid profile of BSFLM also exert synergistic beneficial effects.

Meat quality is a key determinant of consumer acceptance and economic value. Dietary BSFLM improved several meat quality attributes of breast muscle, with the most pronounced effects at 15% inclusion. Breast muscle pH was not significantly affected [33]. The 15% BSFLM group exhibited significantly increased redness (a* value) and decreased yellowness (b* value) compared with the control. The elevated a* value likely results from the deposition of insect-derived pigments in intramuscular fat, as previously noted in broilers [34], while the reduced b* may reflect antioxidant protection of myocyte membranes. Shear force and 48 h drip loss were significantly lowered, indicating enhanced tenderness and water-holding capacity, both of which are key indicators of premium meat [35]. Regarding nutritional composition, 15% BSFLM significantly elevated intramuscular fat (IMF) content, which is well recognized to improve meat tenderness and flavor [36]. The increase in IMF likely contributed to the reduction in shear force. Amino acid profiling revealed that 15% BSFLM significantly increased methionine and leucine contents, and 10% BSFLM increased aspartic acid, with no detrimental effects on the overall amino acid composition. The elevated histidine content may contribute to the reduction in cooking loss, attributable to its free radical-scavenging activity [37]. Meanwhile, the increased proline content may be associated with the higher 24 h pH value, which is likely related to its role in collagen cross-linking and postmortem acidification delay [38,39]. Muscle fiber diameter, cross-sectional area, and density were unaffected, suggesting that BSFLM up to 15% does not adversely affect muscle fiber development.

The fatty acid profile of meat is closely linked to its nutritional value and oxidative stability. BSFLM supplementation selectively modified breast muscle fatty acid composition. Both 10% and 15% BSFLM significantly increased monounsaturated fatty acids (MUFAs), particularly oleic acid (C18:1n9c), whereas 10% BSFLM significantly reduced C20:2 (n-6 PUFA). The total SFAs, total PUFAs, and unsaturated/saturated fatty acid ratio remained unchanged among groups. The increase in MUFAs is consistent with studies in laying hens fed BSFLM [40] and can be directly attributed to the high oleic acid content in BSFL oil (approximately 16.66%), along with the potential elongation and desaturation of medium-chain SFAs such as lauric acid. The pronounced reduction in C20:2 exclusively in the 10% BSFLM group suggests a dose-dependent modulation of fatty acid metabolism, possibly through changes in the dietary SFA/PUFA ratio that affect hepatic desaturase and elongase activities. Collectively, these data indicate that BSFLM can improve the nutritional profile of chicken meat by increasing beneficial MUFAs while maintaining overall fatty acid homeostasis. Moreover, BSFLM reduced MDA accumulation in breast muscle, attenuating post-slaughter lipid peroxidation and protein carbonylation, which helps preserve water-holding capacity and textural quality [41,42]. This effect is plausibly mediated by the Nrf2-dependent antioxidant system.

The gastrointestinal tract is the principal site of nutrient digestion and absorption, and its health is fundamental to poultry growth performance and physiological homeostasis. BSFLM supplementation improved multiple parameters of intestinal health. BSF inclusion significantly increased α-amylase activity in the small intestine, with increases in duodenal lipase and jejunal/ileal trypsin activities. Elevated digestive enzyme activity enhances nutrient breakdown and utilization, which may explain the improved feed efficiency. The parallel trends in serum GSH-Px and T-SOD activities and intestinal lipase activity may support the hypothesis that redox homeostasis contributes to the maintenance of digestive enzyme function [43]. Regarding intestinal morphology, BSFLM significantly increased villus height in the duodenum and ileum without affecting intestinal length. Taller villi indicate a more developed absorptive epithelium [44], consistent with prior findings in layers and broilers [32,40]. The absence of intestinal shortening, in contrast to a report in rutin chickens [45], might be due to chitin degradation during BSFLM processing. For intestinal barrier function, 10% and 15% BSFLM significantly reduced serum diamine oxidase (DAO) activity, a biomarker of mucosal injury [46], suggesting alleviation of intestinal damage. Furthermore, BSFLM significantly upregulated the tight junction proteins Occludin and Claudin-1 in the jejunum, which are essential for regulating paracellular permeability and preventing pathogen translocation [47], as also observed in pigs fed BSFLM products [48,49]. Intestinal antioxidant enzyme activities were also elevated, further supporting gut health.

The cecal microbiota plays a vital role in maintaining intestinal homeostasis, modulating nutrient metabolism, and orchestrating immune responses in poultry. BSFLM induced a restructuring of the cecal microbial community without altering α-diversity. At the phylum level, Bacteroidota and Firmicutes predominated across all groups, collectively accounting for over 88% of relative abundance, which aligns with the core microbiota of poultry. The 10% BSFLM group showed the highest Firmicutes and lowest Bacteroidota proportions, although the F/B ratio did not differ significantly. Euryarchaeota abundance increased with rising BSFLM inclusion, indicating possible shifts in intestinal hydrogen metabolism. At the genus level, 15% BSFLM enriched *Bacteroides*, a polysaccharide-degrading genus capable of utilizing chitin and dietary fiber to produce short-chain fatty acids that benefit barrier function. Conversely, *Rikenellaceae_RC9_gut_group* decreased in BSFLM-supplemented groups, a change that requires further evaluation in conjunction with immune markers. LEfSe analysis identified dose-specific enrichment of functional bacteria: 5% BSFLM enriched *Bifidobacterium*, a well-known probiotic with mucosal protective and anti-inflammatory properties; 10% BSFLM enriched *Oscillospiraceae*, which is associated with anti-inflammatory activity; and 15% BSFLM enriched *Enterococcus*, a conditional pathogen that might be linked to high dietary protein load. These results demonstrate that BSFLM exerts dose-dependent modulation of the cecal microbiota, emphasizing the need to optimize inclusion levels to balance nutrient utilization efficiency and intestinal microecological safety. Several limitations of this study should be acknowledged. First, only female Wenchang chickens were used in the trial, so the findings may not be fully generalizable to male birds. Second, the feeding trial had a relatively short duration, and the long-term effects of BSFLM supplementation remain to be clarified. Third, six replicate pens per treatment were used for most analyses, and the sample size was relatively limited; larger-scale trials are needed to further validate the results. Fourth, only objective meat quality parameters were determined, and sensory evaluation of meat palatability was not conducted. Finally, the long-term safety of high-dose BSFLM inclusion on intestinal microecology requires further systematic investigation.

In conclusion, dietary inclusion of full-fat BSFLM up to 15% confers multiple benefits in Wenchang chickens, including improved feed efficiency, activation of the Nrf2–Keap1 antioxidant signaling pathway, enhanced meat quality and nutritional value, and promotion of intestinal health through augmented digestive capacity, reinforced barrier integrity, and favorable restructuring of the cecal microbiota. Optimal improvements in meat quality were observed at the 15% inclusion level, whereas 5% inclusion most effectively promoted the enrichment of intestinal probiotics. These findings provide comprehensive scientific evidence supporting the use of BSFLM as a sustainable alternative protein source in Wenchang chicken production. Future studies are warranted to elucidate the specific molecular mechanisms underlying BSFLM’s regulatory effects on fatty acid metabolism and microbiota–host crosstalk and to evaluate the long-term effects of high-dose BSFLM on growth performance and intestinal microecological safety.

## 5. Conclusions

Dietary supplementation with 5% to 15% full-fat BSFLM as a soybean meal substitute exerts dose-dependent beneficial effects on Wenchang chickens. Inclusion of 15% BSFLM significantly improves growth performance, feed efficiency, and meat quality, while 5% inclusion optimizes intestinal probiotic composition. These beneficial effects are associated with enhanced antioxidant capacity via activation of the Nrf2-Keap1 pathway, improved intestinal barrier function, and remodeled cecal microbiota. Nevertheless, the enrichment of Enterococcus observed in the 15% BSFLM group raises safety concerns and warrants long-term feeding assessment. This study provides scientific evidence for BSFLM as a sustainable alternative protein source in high-quality native broiler production, supporting the green development of the poultry industry.

## Figures and Tables

**Table 1:** Effects of dietary BSFLM on growth performance of Wenchang chickens ^1^.

Items	Groups				SEM	*p*-Value
	H0	H5	H10	H15		
Initial weight, g	524.13	523.75	525.47	526.62	0.57	0.329
Final weight, g	1343.86 ^b^	1389.05 ^ab^	1399.17 ^ab^	1433.74 ^a^	16.04	0.023
Average daily gain (ADG), g	19.52	20.36	20.32	21.27	0.31	0.122
Average daily feed intake (ADFI), g	94.15	92.84	92.04	89.7	0.81	0.577
F/G (ADFI/ADG)	4.83 ^a^	4.56 ^ab^	4.5 ^ab^	4.23 ^b^	0.11	0.009
Feed prices (RMB/kg)	2.77	2.62	2.47	2.33	0.08	-
Unit increased feed cost (RMB/kg)	13.36 ^a^	11.95 ^b^	11.12 ^b^	9.84 ^c^	0.64	<0.001

^1^ In the same row, values with no letter or the same letter superscripts show no significant differences (*p* > 0.05), while those with different letter superscripts show significant differences (*p* < 0.05); *n* = 6. H0, blank control group, Wenchang chickens were fed a diet without BSFLM supplementation; H5, H10, and H15, Wenchang chickens were fed a diet supplemented with 5%, 10%, and 15% BSFLM, respectively.

**Table 2:** Effects of dietary BSFLM on slaughter performance of Wenchang chickens ^1^.

Items	Groups				SEM	*p*-Value
	H0	H5	H10	H15		
Live weight (g)	1397.19	1402.14	1509.16	1435.64	22.37	0.056
Dressing percentage (%)	89.31	90.12	90.12	90.02	0.31	0.173
Eviscerated yield percentage (%)	59.00	60.43	62.28	61.18	0.78	0.270
Chest muscle percentage (%)	10.43	10.54	10.66	10.55	0.05	1.000
Leg muscle percentage (%)	19.12	19.03	17.67	18.38	0.38	0.448
Body fat percentage (%)	7.02	7.53	7.32	8.14	0.12	0.136
Wing percentage (%)	11.74	11.53	11.56	11.62	0.05	0.847
Liver indices	2.20	2.11	1.96	1.95	0.06	0.085
Spleen index	0.29	0.22	0.22	0.21	0.02	0.168
Thymus index	0.39	0.45	0.41	0.45	0.05	0.610
Fowls’ sac index	0.26	0.27	0.22	0.22	0.01	0.399
Kidney index	0.48	0.51	0.51	0.49	0.04	0.902

^1^ n = 6. H0, blank control group, Wenchang chickens were fed a diet without BSFLM supplementation; H5, H10, and H15, Wenchang chickens were fed a diet supplemented with 5%, 10%, and 15% BSFLM, respectively.

**Table 3:** Effects of dietary BSFLM on meat quality of Wenchang chickens ^1^.

Items	Groups				SEM	*p*-Value
	H0	H5	H10	H15		
L*	52.70	53.19	53.71	52.18	0.28	0.462
a*	4.24 ᵇ	5.10 ᵃᵇ	5.31 ᵃᵇ	5.61 ᵃ	0.25	0.034
b*	5.69	5.63	5.30	5.23	0.10	0.590
pH_45 min_	6.50	6.32	6.39	6.40	0.03	0.139
pH_24 h_	6.07	6.02	6.07	6.07	0.01	0.302
Shear force (N)	73.50 ᵃ	72.60 ᵃᵇ	62.31 ᵃᵇ	59.28 ᵇ	3.11	0.043
Drip loss (% 24 h)	3.52	3.81	3.61	2.67	0.48	0.516
Drip loss (% 48 h)	6.02 ᵃ	5.61 ᵃᵇ	4.24 ᵃᵇ	4.47 ᵇ	0.51	0.048
Cooking loss (%)	15.04 ᵃ	15.52 ᵃᵇ	12.45ᵇ	12.56 ᵇ	0.74	<0.010

^1^ In the same row, values with no letter or the same letter superscripts show no significant difference (*p* > 0.05), while those with different letter superscripts show significant differences (*p* < 0.05); *n* = 6. H0, blank control group, Wenchang chickens were fed a diet without BSFL supplementation; H5, H10, and H15, Wenchang chickens were fed a diet supplemented with 5%, 10%, and 15% BSFL, respectively. L*, a*, and b* represent lightness, redness, and yellowness, respectively.

**Table 4:** Effects of dietary BSFLM on amino acid composition of Wenchang chickens ^1^.

Items	Groups				SEM	*p*-Value
	H0	H5	H10	H15		
Ala	54.24	39.39	44.60	51.23	2.89	0.100
Met	7.87 ^b^	7.55 ^b^	11.08 ^ab^	13.15 ^a^	1.16	0.012
Arg	18.17	12.96	15.69	19.16	1.20	0.185
Asp	8.01 ^b^	9.44 ^b^	19.34 ^a^	14.71 ^ab^	2.24	0.004
Cys	0.26	0.23	0.26	0.24	0.01	0.213
Glu	67.59	74.53	73.95	82.66	2.68	0.397
Gly	116.29	108.36	127.45	115.99	3.40	0.995
His	7.19	12.64	12.66	12.47	1.17	0.135
Ile	13.26	9.43	10.57	13.99	0.94	0.120
Leu	15.04 ^b^	17.73 ^ab^	21.26 ^ab^	25.17 ^a^	1.90	0.022
Lys	90.12	77.66	96.73	86.86	3.44	0.424
Phe	15.98	16.34	16.92	19.30	0.65	0.668
Pro	9.74	9.08	10.46	10.78	0.33	0.365
Ser	16.64	19.57	20.66	18.53	0.74	0.267
Thr	14.84	16.78	18.10	19.59	0.87	0.686
Tyr	24.67	23.65	25.85	34.86	2.23	0.104
Val	8.95	10.63	12.90	12.32	0.77	0.534

^1^ In the same row, values with no letter or the same letter superscripts show no significant difference (*p* > 0.05), while those with different letter superscripts show significant differences (*p* < 0.05); *n* = 6. H0, blank control group, Wenchang chickens were fed a diet without BSFLM supplementation; H5, H10, and H15, Wenchang chickens were fed a diet supplemented with 5%, 10%, and 15% BSFLM, respectively.

**Table 5:** Effects of dietary BSFLM on fatty acid composition of Wenchang chickens ^1^.

Items	Groups				SEM	*p*-Value
	H0	H5	H10	H15		
C6:0	3.19	3.12	3.09	3.18	0.10	0.897
C8:0	1.42	1.29	1.33	1.20	0.14	0.767
C10:0	5.37	5.32	5.22	5.23	0.18	0.933
C14:0	0.94	0.89	0.79	0.80	0.09	0.630
C16:0	6.47	6.46	6.37	6.39	0.17	0.963
C17:0	1.06	1.04	0.96	0.93	0.10	0.789
C18:0	14.44	14.92	14.47	14.53	0.14	0.143
C20:0	0.20	0.22	0.19	0.15	0.03	0.473
C21:0	0.31	0.26	0.24	0.31	0.04	0.427
C22:0	1.21	1.15	1.15	1.16	0.05	0.765
C14:1n5	0.90	0.85	0.86	0.94	0.05	0.722
C15:1n5	0.39	0.39	0.38	0.37	0.06	0.983
C16:1n7	3.39	3.32	3.34	3.39	0.07	0.827
C17:1n7	0.32	0.28	0.29	0.29	0.03	0.920
C20:1n9	0.19	0.10	0.14	0.20	0.02	0.044
C22:1n9	0.33	0.28	0.31	0.31	0.03	0.769
C18:1n9c	41.77 ^c^	42.50 ^bc^	43.75 ^a^	43.37 ^ab^	0.23	0.001
C18:2n6c	0.93	1.08	0.99	0.97	0.07	0.467
C18:3n6	1.66	1.56	1.51	1.50	0.14	0.871
C20:2	1.35 ^a^	1.27 ^a^	1.00 ^b^	1.28 ^a^	0.05	0.002
C20:3n6	4.46	4.27	4.27	4.30	0.13	0.733
C20:4n6	5.51	5.37	5.24	5.29	0.13	0.536
C20:5n3	1.53	1.49	1.61	1.45	0.07	0.474
C22:2n6	0.89	0.81	0.80	0.77	0.10	0.891
C22:6n3	1.80	1.78	1.71	1.71	0.09	0.847
SFA	34.60	34.65	33.81	33.89	0.58	0.640
USFA	65.40	65.35	66.20	66.12	0.45	0.429
MUFA	47.29^c^	47.73 ^bc^	49.06 ^a^	48.86 ^ab^	0.30	0.001
PUFA	18.12	17.62	17.14	17.25	0.31	0.144
USFA/SFA	0.53	0.53	0.51	0.51	0.01	0.329

^1^ In the same row, values with no letter or the same letter superscripts show no significant difference (*p* > 0.05), while those with different letter superscripts show significant differences (*p* < 0.05); *n* = 6. H0, blank control group, Wenchang chickens were fed a diet without BSFLM supplementation; H5, H10, and H15, Wenchang chickens were fed a diet supplemented with 5%, 10%, and 15% BSFLM, respectively.

## Data Availability

The original contributions presented in the study are included in the article; further inquiries can be directed to the corresponding authors.

## Supplementary Materials

The following supporting information can be downloaded at https://www.mdpi.com/article/10.3390/ani16162608/s1, Table S1: Composition of black soldier fly larvae meal (air-dry basis); Table S2: Amino acid content of black soldier fly larvae meal (mg/mL); Table S3: Test diet composition and nutrient level (air-dry basis) (%).

## Abbreviations

The following abbreviations are used in this manuscript:

BSFLM	Black soldier fly larvae meal
BW	Body weight
ADG	Average daily gain
ADFI	Daily feed intake
F/G	Feed-to-gain ratio
T-AOC	Total antioxidant capacity
GSH-Px	Glutathione peroxidase
T-SOD	Total superoxide dismutase
MDA	Malondialdehyde
SFA	Saturated fatty acid
MUFA	Monounsaturated fatty acid
PUFA	Polyunsaturated fatty acid
H&E	Hematoxylin and eosin
OTUs	Operational taxonomic units
ANOVA	One-way analysis of variance
HSD	Honestly significant difference
PCoA	Principal coordinate analysis
PERMANOVA	Permutational multivariate analysis of variance
LEfSe	Linear discriminant analysis effect size
DAO	Diamine oxidase
ROS	Reactive oxygen species
ARE	Antioxidant response element
IMF	Intramuscular fat
VH	Villus height
CD	Crypt depth
VH/CD	Villus height-to-crypt depth ratio

## References

[1] Cheng Z., Jia Y., Bai Y., Zhang T., Ren K., Zhou X., Zhai Y., Shen X., Hong J. (2023). Intensifying the environmental performance of chicken meat production in China: From perspective of life cycle assessment. J. Clean. Prod..

[2] Wiedemann S.G., McGahan E.J., Murphy C.M. (2017). Resource use and environmental impacts from Australian chicken meat production. J. Clean. Prod..

[3] McAuliffe G.A., Takahashi T., Lee M.R.F. (2018). Framework for life cycle assessment of livestock production systems to account for the nutritional quality of final products. Food Energy Secur..

[4] Tantiyasawasdikul V., Chomchuen K., Loengbudnark W., Chankitisakul V., Boonkum W. (2023). Comparative study and relationship analysis between purine content, uric acid, superoxide dismutase, and growth traits in purebred and crossbred Thai native chickens. Front. Vet. Sci..

[5] Qi Q., Hu C., Zhang H., Sun R., Liu Q., Ouyang K., Xie Y., Li X., Wu W., Liu Y. (2023). Dietary Supplementation with Putrescine Improves Growth Performance and Meat Quality of Wenchang Chickens. Animals.

[6] He Y., Li J., Wang F., Na W., Tan Z. (2023). Dynamic Changes in the Gut Microbiota and Metabolites during the Growth of Hainan Wenchang Chickens. Animals.

[7] Biasato I., Demarco M., Rotolo L., Renna M., Lussiana C., Dabbou S., Capucchio M.T., Biasibetti E., Costa P., Gai F. (2016). Effects of dietary *Tenebrio Molitor* meal inclusion in free-range chickens. J. Anim. Physiol. Anim. Nutr..

[8] Oluokun J.A. (2010). Upgrading the nutritive value of full-fat soyabeans meal for broiler production with either fishmeal or black soldier fly larvae meal (*Hermitia illucens*). Niger. J. Anim. Sci..

[9] Ramos-Elorduy J., Moreno J.M.P., Prado E.E., Perez M., Otero J., Guevara O. (1997). Nutritional Value of Edible Insects from the State of Oaxaca, Mexico. J. Food Compos. Anal..

[10] Biasato I., Gassco L., De Marco M., Rotolo L., Dabbou L., Biasibetti M., Tarantola M., Sterpone L., Cavallarin L., Gai F. (2018). Yellow mealworm larvae (*Tenebrio molitor*) inclusion in diets for male broiler chickens: Effects on growth performance, gut morphology, and histological findings. Poult. Sci..

[11] Alegbeleye W.O., Obasa S.O., Olude O.O., Otubu K., Jimoh K. (2012). Preliminary evaluation of the nutritive value of the variegated grasshopper (*Zonocerus variegatus* L.) for African catfish *Clarias gariepinus* (Burchell. 1822) fingerlings. Aquac. Res..

[12] (2004). Feeding Standard of Chicken.

[13] (2015). Objective Evaluation Method for Eating QUALITY of Meat.

[14] (2016). National Food Safety Standard—Determination of Moisture in Foods.

[15] (2016). National Food Safety Standard—Determination of Ash in Foods.

[16] (2016). National Food Safety Standard—Determination of Protein in Foods.

[17] (2016). National Food Safety Standard—Determination of Fat in Foods.

[18] Liu Y.X., Kong F.B., Tan Y.L., Duan Y., Yin H., Hu C., Guoyao F. (2016). Co-dependence of genotype and die tary protein intake to affect expression on amino acid/peptide transporters in porcine skeletal muscle. Amino Acids.

[19] Ramesh Bahadur B., Keshav B., Sandesh P., Xiao Y., Bidur P., Sudhagar M., Dongyi W., Lilong C. (2024). Sustainable poultry farming practices: A critical review of current strategies and future prospects. Poult. Sci..

[20] Djordjevic M., Idriss L., Chaibou M., Johann D., Quynh C. (2008). Effects of substitution of fish meal with fresh and dehydrated larvae of the house fly (*Musca domestica* L) on productive performance and health of broilers. Acta Vet..

[21] Sedgh-gooya S., Torki M., Darbemamieh M., Hassan K., Mohammad A., Alireza A. (2021). Yellow mealworm, *Tenebrio molitor* (Col: Tenebrionidae), larvae powder as dietary protein sources for broiler chickens: Effects on growth performance, carcass traits, selected intestinal microbiota and blood parameters. J. Anim. Physiol. Anim. Nutr..

[22] Jin X.H., Heo P.S., Hong J.S., Kim N.J., Kim Y.Y. (2016). Supplementation of Dried Mealworm (*Tenebrio molitor* larva) on Growth Performance, Nutrient Digestibility and Blood Profiles in Weaning Pigs. Asian-Australas. J. Anim. Sci..

[23] Schiavone A., Demarco M., Martínez S., Dabbou S., Renna M., Madrid J., Hernandez F., Rotolo L., Costa P., Gai F. (2017). Nutritional value of a partially defatted and a highly defatted black soldier fly larvae meal for broiler chickens: Apparent nutrient digestibility, apparent metabolizable energy and apparent ileal amino acid digestibility. J. Anim. Sci. Biotechnol..

[24] Newton G.L., Booram C.V., Barker R.W., Josefa M., Fuensanta H., Luca R., Pierluca C., Francesco G., Laura G. (1977). Dried Hermetia Illucens Larvae Meal as a Supplement for Swine. J. Anim. Sci..

[25] Sara O., Ilaria B., Arianna I., Miha P., Dominik D., Elena C., Maria T., Marco M., Stefania B., Raffaella B. (2021). Black soldier fly and yellow mealworm live larvae for broiler chickens: Effects on bird performance and health status. J. Anim. Physiol. Anim. Nutr..

[26] Schiavone A., Dabbous S., Petacci M., Zampiga M., Sirri F., Biasato I., Gai F., Gasco L. (2019). Black soldier fly defatted meal as a dietary protein source for broiler chickens: Effects on carcass traits, breast meat quality and safety. Animal.

[27] Al-qazzaz M., Samsudin A.A., Idris L.H., Ismail D., Akit H. (2021). Dietary Replacement with Food Waste and Black Soldier Fly Larvae Supplementation Improved Growth Performance, Nutrient Digestibility and Intestinal Microbial Population in Broilers. Int. J. Agric. Biol..

[28] Chen Y., Gu Y., Zhao H., Zhang H., Zhou Y. (2020). Effects of graded levels of dietary squalene supplementation on the growth performance, plasma biochemical parameters, antioxidant capacity, and meat quality in broiler chickens. Poult. Sci..

[29] Chu X., Li M., Wang G., Wang K., Shang R., Wang Z., Li L. (2020). Evaluation of the Low Inclusion of Full-Fatted *Hermetia illucens* Larvae Meal for Layer Chickens: Growth Performance, Nutrient Digestibility, and Gut Health. Front. Vet. Sci..

[30] Lee J., Kim Y.M., Parky K., Young-Cheol Y., Bock-Gie J., Bong-Joo L. (2018). Black soldier fly (*Hermetia illucens*) larvae enhances immune activities and increases survivability of broiler chicks against experimental infection of Salmonella Gallinarum. J. Vet. Med. Sci..

[31] Marta G., Sihem D., Manuela C., Ilaria B., Francesco G., Laura G., Francesco P., Patrizio O., Stefania B., Iveta P. (2019). Effects of the Dietary Inclusion of Partially Defatted Black Soldier Fly (*Hermetia illucens*) Meal on the Blood Chemistry and Tissue (Spleen, Liver, Thymus, and Bursa of Fabricius) Histology of Muscovy Ducks (*Cairina moschata domestica*). Animals.

[32] Jin S., Yang H., Jiao Y., Pang Q., Wang Y., Wang M., Shan A., Feng X. (2021). Dietary Curcumin Alleviated Acute Ileum Damage of Ducks (*Anas platyrhynchos*) Induced by AFB1 through Regulating Nrf2-ARE and NF-κB Signaling Pathways. Foods.

[33] Ma C., Zhang W., Zhang J., Zhou L., Xing L., Liu R. (2026). Postmortem energy metabolism and meat quality development: Advances in basic pathways, endogenous regulatory factors, and exogenous management strategies. J. Adv. Res..

[34] Cullere M., Tasoniero G., Giaccone V., Miotti-Scapin R., Claeys E., De Smet S., Zotte A.D. (2016). Black soldier fly as dietary protein source for broiler quails: Apparent digestibility, excreta microbial load, feed choice, performance, carcass and meat traits. Animal.

[35] Fernandez X., Monin G., Talmant A., Mourot J., Lebret B. (1999). Influence of intramuscular fat content on the quality of pig meat—2. Consumer acceptability of m. longissimus lumborum. Meat Sci..

[36] Kilburn J., Edwards H.W. (2001). The response of broilers to the feeding of mash or pelleted diets containing maize of varying particle sizes. Br. Poult. Sci..

[37] Sun Y., Liu C., Li Y., Li D., Shi L., Chen J. (2023). Effect of Cage and Floor Housing Systems on Muscle Fiber Characteristics, Carcass Characteristics, and Meat Quality of Slow-Growing Meat-Type Chickens. Agriculture.

[38] Zeugolis D.I., Raghunath M. (2011). 2.215—Collagen: Materials Analysis and Implant Uses. Compr. Biomater..

[39] Samaneh Z., Bahrami A., Khan M.T.H., Munoz-Munoz J., Garcia-Molina F., Garcia-Canovasd F., Sabourye A.A. (2019). A comprehensive review on tyrosinase inhibitors. J. Enzym. Inhib. Med. Chem..

[40] Cutrignelli M.I., Messina M., Tulli F., Basilio R., Ike O., Laura G., Rosa L., Fulvia B. (2018). Evaluation of an insect meal of the Black Soldier Fly (*Hermetia illucens*) as soybean substitute: Intestinal morphometry, enzymatic and microbial activity in laying hens. Res. Vet. Sci..

[41] Papuc C., Goran G.V., Predescu C.N., Nicorescu V. (2017). Mechanisms of Oxidative Processes in Meat and Toxicity Induced by Postprandial Degradation Products: A Review. Compr. Rev. Food Sci. Food Saf..

[42] Falowo A.B., Fayemi P.O., Muchenje V. (2014). Natural antioxidants against lipid–protein oxidative deterioration in meat and meat products: A review. Food Res. Int..

[43] Zhang L.Y., Peng Q.Y., Liu Y.R., Ma Q., Zhang J., Guo Y., Xue Z., Zhao L. (2021). Effects of oregano essential oil as an antibiotic growth promoter alternative on growth performance, antioxidant status, and intestinal health of broilers. Poult. Sci..

[44] Chamorro S., Romero C., Brenes A., Fernando S., Begoña B., Agustín V., Ignacio A. (2019). Impact of a sustained consumption of grape extract on digestion, gut microbial metabolism and intestinal barrier in broiler chickens. Food Funct..

[45] Chen G., Zhang K., Tang W., Li Y., Pang J., Yuan X., Song X., Jiang L., Yu X., Zhu X. (2023). Feed nutritional composition affects the intestinal microbiota and digestive enzyme activity of black soldier fly larvae. Front. Microbiol..

[46] Fukudome I., Kobayashi M., Dabanaka K., Hiromichi M., Ken O., Takehiro O., Ryoko B., Nana K., Koji O., Mamoru F. (2013). Diamine oxidase as a marker of intestinal mucosal injury and the effect of soluble dietary fiber on gastrointestinal tract toxicity after intravenous 5-fluorouracil treatment in rats. Med. Mol. Morphol..

[47] Carrasco J.M.D., Casanova N.A., Miyakawa M.E. (2019). Microbiota, Gut Health and Chicken Productivity: What Is the Connection?. Microorganisms.

[48] Harhaj N.S., Antonetti D.A. (2004). Regulation of tight junctions and loss of barrier function in pathophysiology. Int. J. Biochem. Cell Biol..

[49] Yu M., Li Z., Chen W., Rong T., Wang G., Ma X. (2019). *Hermetia illucens* larvae as a potential dietary protein source altered the microbiota and modulated mucosal immune status in the colon of finishing pigs. J. Anim. Sci. Biotechnol..

