# Supplementary material for: Dietary Black Soldier Fly Larvae Meal Improves Growth Performance and Meat Quality Associated with Enhanced Antioxidant Capacity and Intestinal Health in Wenchang Chickens
Source: Animals (Basel). 2026 Aug 20;16(16):2608. doi: 10.3390/ani16162608 (PMC13509808; doi:10.3390/ani16162608)
Supplement: Supplementary file 1 [file animals-16-02608-s001.zip › animals-4497521-supplementary.pdf]

Table S1 Composition of black soldier fly larvae meal (air-dry basis)

Item	Content (%)
Moisture	2.98
Crude protein	36.00
Crude fat	38.09
Crude ash	10.39
Calcium	2.82
Phosphorus	0.79

Table S 2 Amino acid content of black soldier fly larvae meal (mg/mL)

Item	Content
Essential AA	
Leu	62.07
Met	10.18
Arg	238.44
Lys	280.51
Phe	22.63
Thr	67.41
Val	117.64
His	229.48
Ile	168.71
Non-essential AA	
Asp	42.94
Cys	0.46
Glu	501.75
Pro	763.13
Ser	50.25
Gly	2544.25
Tyr	426.99
Ala	758.46

Table S3 Test diet composition and nutrient level (air-dry basis) (%)

Item	H0	H5	H10	H15
Corn	75.30	73.50	71.80	70.00
Soybean meal	18.20	15.00	11.70	8.50
Wheat bran	3.50	3.50	3.50	3.50
Black soldier fly	0.00	5.00	10.00	15.00
Calcium phosphate (anhydrous)	1.10	1.10	1.10	1.10
Stone powder	1.10	1.10	1.10	1.10
Table salt	0.20	0.20	0.20	0.20
Premix[1]	0.50	0.50	0.50	0.50
Mildew-proof agent	0.10	0.10	0.10	0.10
Total	100.00	100.00	100.00	100.00
Nutritional level[3]				
Metabolizable energy (MJ/kg)	12.24	12.24	12.27	12.28
Crude protein	15.10	15.05	15.10	15.10
Crude fat	3.20	4.99	6.78	8.57
Calcium	0.83	0.95	1.08	1.21
Total phosphorus	0.60	0.61	0.62	0.64
Lysine	0.76	0.70	0.73	0.75
Methionine	0.27	0.26	0.27	0.28

Note:

1. The premix provides per kilogram of diet: VA 33000 IU; VD_3 9000 IU; VE 10 IU; VK 0.5 mg; VB1 3.00 mg; VB2 8.00 mg; VB6 4.40 mg; VB12 0.012 mg; niacin 30 mg; calcium pantothenate 15 mg; folic acid 0.75 mg; biotin 0.20 mg; choline 1100 mg; iron 80 mg; copper 8 mg; zinc 80 mg; manganese 80 mg; iodine 0.7 mg; selenium 0.3 mg.

2. H0 is the control group, corn-soybean meal-based basal diet; H5, H10, and H15 represent diets in which soybean meal is partially replaced by nitrogen at levels of 5%, 10%, and 15%, respectively.

3. Except for metabolizable energy, the nutrient levels are the measured values.
